# The role of the infectious disease physician in abdominal wall repair using prosthetic material after clean-contaminated surgery

**DOI:** 10.1186/1471-2334-14-S7-P81

**Published:** 2014-10-15

**Authors:** Daniel Ion, Răzvan Vasile Stoian, Simona Elena Albu, Alexandra Bolocan, Dan Nicolae Păduraru

**Affiliations:** 1 University Emergency Hospital of Bucharest, Romania

## Background

Use of antibiotic prophylaxis when prosthetic implants are present at the abdominal wall was a controversial decision. It is a delicate matter since parietal prosthetics frequently associate surgical visceral interventions (cholecystectomy, appendectomy, enterectomy/enterorrhaphy, and colectomy).

## Methods

This study was carried out in the Surgery and Emergency Clinic III of the University Emergency Hospital Bucharest. It is based on a 5 year experience (2010-2014), retrospective evaluation on 224 incision hernias resolved with prosthetic material, of which 216 in a clean environment and 28 in a clean-contaminated environment. For all the cases the antibiotic prophylaxis management was decided by a mixed team: general surgeon and infectious disease physician.

## Results

Results plead for the rational and selective use of antibiotics for the patients that require prosthetic material and draw attention to the extremely favorable results of the collaboration between different specialty physicians.

## Conclusion

Even though the initial dogma was that all alloplastic grafts need “protection” provided by the antibiotics, recent approaches tend to be more selective and permissive.

